# The fungus *Neurospora crassa *displays telomeric silencing mediated by multiple sirtuins and by methylation of histone H3 lysine 9

**DOI:** 10.1186/1756-8935-1-5

**Published:** 2008-11-03

**Authors:** Kristina M Smith, Gregory O Kothe, Cindy B Matsen, Tamir K Khlafallah, Keyur K Adhvaryu, Melissa Hemphill, Michael Freitag, Mohammad R Motamedi, Eric U Selker

**Affiliations:** 1 Institute of Molecular Biology and Department of Biology, University of Oregon, Eugene, OR 97403, USA; 2 Department of Cell Biology, Harvard Medical School, Boston, MA 02115, USA

## Abstract

**Background:**

Silencing of genes inserted near telomeres provides a model to investigate the function of heterochromatin. We initiated a study of telomeric silencing in *Neurospora crassa*, a fungus that sports DNA methylation, unlike most other organisms in which telomeric silencing has been characterized.

**Results:**

The selectable marker, *hph*, was inserted at the subtelomere of Linkage Group VR in an *nst-1 *(n*eurospora *s*ir *t*wo*-1) mutant and was silenced when *nst-1 *function was restored. We show that NST-1 is an H4-specific histone deacetylase. A second marker, *bar*, tested at two other subtelomeres, was similarly sensitive to *nst-1 *function. Mutation of three additional SIR2 homologues, *nst-2*, *nst-3 *and *nst-5*, partially relieved silencing. Two genes showed stronger effects: *dim-5*, which encodes a histone H3 K9 methyltransferase and *hpo*, which encodes heterochromatin protein-1. Subtelomeres showed variable, but generally low, levels of DNA methylation. Elimination of DNA methylation caused partial derepression of one telomeric marker. Characterization of histone modifications at subtelomeric regions revealed H3 trimethyl-K9, H3 trimethyl-K27, and H4 trimethyl-K20 enrichment. These modifications were slightly reduced when telomeric silencing was compromised. In contrast, acetylation of histones H3 and H4 increased.

**Conclusion:**

We demonstrate the presence of telomeric silencing in Neurospora and show a dependence on histone deacetylases and methylation of histone H3 lysine 9. Our studies also reveal silencing functions for DIM-5 and HP1 that appear independent of their role in *de novo *DNA methylation.

## Background

Linear chromosomes pose a problem for replication of the terminal section of the DNA strand with a 5' end. The problem is solved in most eukaryotes by the addition of repeated sequences to the chromosome ends [1]. Thus the budding yeast *Saccharomyces cerevisiae *sports TG_1–3 _repeats on the ends of its chromosomes [2], while telomeres of both the filamentous fungus *Neurospora crassa *and humans comprise TTAGGG repeats [3,4]. Drosophila's chromosome ends are capped by arrays of retrotransposons and the adjacent subtelomeric DNA consists of repetitive elements called telomere-associated sequences (TAS) [4]. TAS appear cytologically condensed (that is, heterochromatic) [5] and confer silencing on nearby genes, apparently because of spreading of silent heterochromatin. This phenomenon, called 'telomeric silencing', or 'telomere position effect' (TPE), was initially discovered and studied using transgenes but it also appears to regulate endogenous subtelomeric genes [6-8].

Telomeric silencing has been demonstrated in organisms ranging from yeasts to humans (reviewed in [9]) but it has been best characterized in *S. cerevisiae*, *Schizosaccharomyces pombe *and *Drosophila melanogaster*, organisms that have no, or very little, DNA methylation. Heterochromatin at *S. cerevisiae *telomeres is nucleated by the telomere repeat binding protein Rap1 [10,11]. Telomeric silencing requires histone deacetylation by the conserved nicotinamide adenine dinucleotide (NAD^+^)-dependent histone deacetylase (HDAC) Sir2p [12], reviewed in [13]. Sir2p is found in a complex with Sir4p, which interacts with Sir3p [14]. While Sir2p homologues (sirtuins) have been found in all eukaryotes examined, Sir3p and Sir4p are restricted to close relatives of *S. cerevisiae*. The fission yeast *S. pombe *lacks Sir3p and Sir4p but employs methylation of histone H3 lysine 9 (K9) plus a Sir2p homologue (Sir2) for silencing [15]. Deacetylation by Sir2 allows methylation of K9 by the Clr4 histone methyltransferase (HKMT), which in turn allows for binding by a homologue of Heterochromatin Protein-1 (HP1), Swi6 [15,16]. Swi6 is recruited to telomeres by interaction with the telomere repeat binding factor Taz1, which interacts with Rap1 and the RNAi-RITS complex [17].

The mechanism of telomeric silencing is largely unknown in plants and animals [1]. Silencing in Drosophila is dependent on telomere length, which depends on HP1 and its interaction with both telomere DNA and methylated H3 K9 [18-21]. One of the five Drosophila *SIR2 *homologues was tested for involvement in telomeric silencing but none was detected [22]. Sequences associated with mammalian telomeres show features of heterochromatin, including DNA methylation, trimethylated H4 K20, trimethylated H3 K9 and bound HP1, but the role of these factors remains to be elucidated [1]. Study of telomeric silencing in humans and mice has been limited, presumably in part because genes involved in heterochromatin formation and DNA methylation are essential in these organisms. We therefore initiated a study of telomeric silencing in *N. crassa*, which has DNA methylation like plants and mammals, but does not depend on it for survival.

A single DNA methyltransferase (DNMT), DIM-2, is responsible for all known DNA methylation in Neurospora [23]. DNA methylation is directed by Neurospora's single HP1 homologue to chromosomal regions in which histone H3 is trimethylated at K9 by the HKMT DIM-5 [24-26]. DNA methylation in Neurospora is found predominantly at transposon relics [27], many of which are scattered throughout the genome but are most concentrated in centromeric and telomeric regions [28-30].

To look for TPE in Neurospora, we inserted transgenes near telomeres in mutants that were defective in candidate silencing genes and were thus predicted to relieve the telomeric silencing. We initially tested several putative sirtuins, which we named *nst-1 *(Neurospora sir two) through *nst-7 *[31]. We found evidence of TPE in Neurospora and found that several but not all of the *nst *genes are involved in silencing. We also tested the involvement of DIM-5, HP1 and DIM-2 to assess the potential influence of DNA methylation on TPE. Finally, we tested the effect of chemical inhibitors of HDACs and DNA methylation. We found evidence for the involvement of DIM-5, HP1 and HDACs in telomeric silencing in Neurospora. Interestingly, we found that the telomeric transgenes and native subtelomeric sequences are lightly methylated and that mutation of *dim-2 *can affect TPE, although not at all telomeres. We conclude that silencing by DNA methylation at non-telomeric chromosomal sites and silencing at telomeres share some components (DIM-5 and HP1) but represent distinct silencing pathways.

## Results

### Inactivation of nst genes

**Figure 1:**
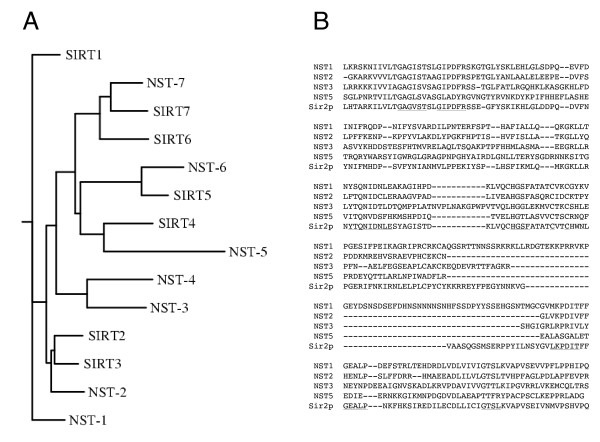
**A) Phylogenetic tree of Sir2 homologues from *N. crassa *(NST-1 through NST-7) and humans (SIRT1 through SIRT7) based on a single CLUSTALW alignment of their putative Sir2 catalytic domains.** B) Alignment of the Sir2 domain from *S. cerevisiae *Sir2p with the corresponding regions of Neurospora sirtuins NST-1, NST-2, NST-3, and NST-5. The most highly conserved regions are underlined in the Sir2p sequence.

To investigate whether Neurospora shows telomeric silencing, we needed to insert a genetic marker in a subtelomeric region and then test its expression in various genetic backgrounds. As the histone H4 K16-specific deacetylase Sir2p is central to telomeric silencing in previously examined eukaryotes and has also been implicated in other forms of silencing [9], we chose to test Neurospora homologues of Sir2p first. The Neurospora genome contains seven genes predicted to encode a protein with the NAD^+^-dependent deacetylase domain typical of the Sir2 family, the same number found in the human genome [32]. We designated these genes *nst-1 *(Neurospora sir two) through *nst-7 *[31]. NST-1 is most closely related to *S. pombe *Sir2p and *S. cerevisiae *Sir2p and Hst1p, two proteins that have partially redundant functions in telomeric silencing [12,33,34]. Neurospora also has close homologues of *S. cerevisiae *Hst3p (NST-4) and Hst4p (NST-3), which both act on acetylated K56 of histone H3 [35] and are required for telomeric silencing in yeast [33]. NST-2 is most closely related to *S. cerevisiae *Hst2p, which is cytosolic and disrupts telomeric silencing when over-expressed [36]. A tree based on a single alignment of the putative Sir2 catalytic domains of the Neurospora and human sirtuins (Sir2 homologues) is shown in Figure 1A. While we cannot assume functional similarities based on this level of sequence similarity, it should be noted that the human sirtuins are not all nuclear histone deacetylases. One study showed localization of the human sirtuins in the nucleus (SIRT1), the cytosol (SIRT2), mitochondria (SIRT3, SIRT4 and SIRT5), heterochromatin (SIRT6), and the nucleolus (SIRT7) [32] (Figure 1A). More recently it was shown that SIRT4 is a mitochondrial ADP-ribosyl transferase [37].

We used RIP (repeat-induced point mutation) [38] to generate strains with nonsense mutations in *nst-1*. One such strain (N1982) served as the initial host for insertion of a telomeric reporter gene (see below). We also employed RIP to generate likely null alleles of several other *nst *genes; altogether, we identified strains with one or more stop codons in, or upstream of, the putative deacetylase domains of *nst-1, -2, -3 *and *-5 *(Figure 1B). The *nst-1*^*RIP*1^sequence (EU869540) introduces a single stop codon at Q255, well within the conserved region of Sir2p, which extends from roughly L197 through Q473. The *nst-2*^*RIP*1^sequence (EU869542) has a mutation in the predicted start codon (ATG to ATA). The next potential start codon is M165, downstream of the region that is predicted to encode the deacetylase catalytic domain. The mutations in *nst-3*^*RIP*1 ^(EU869541) generate a stop codon at Q69. The *nst-5*^*RIP*1 ^sequence (EU872050) contains seven predicted nonsense mutations (at W115, R152, Q163, Q177, Q196, Q203, and R234). Interestingly, unlike the case in *S. cerevisiae *[33], none of these mutations resulted in any significant growth phenotype, even when *nst-1, -3*, and *-5 *were combined (data not shown).

### Silencing of *hph *at Telomere VR

**Figure 2:**
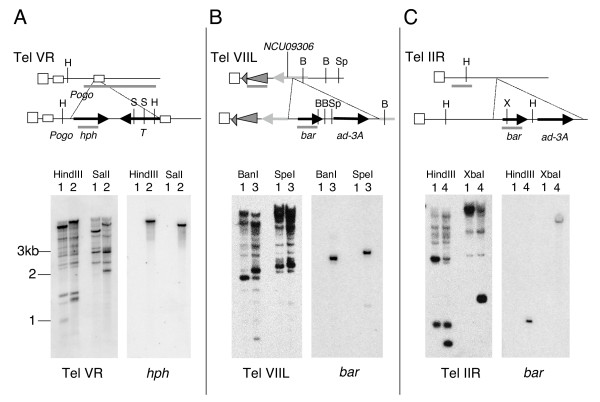
**Engineered subtelomeric markers.** A-C) Top of each panel shows a cartoon map (not to scale) of inserted selectable markers at telomeres VR (contig 7.37, panel A), VIIL (contig 7.251, panel B) and IIR (contig 7.77, panel C). Vertical striped bars represent telomeric (TTAGGG)_n _repeats and diagonal striped bars in A represent *Pogo *LTRs [29]. Gray triangles on Tel VIIL (B) represent CenVII repeat element (194–280 bp from end) and a second unnamed repeat element (280–1077 bp from end). Southern blots of DNA from strains 1 (N150; WT), 2 (N3120; Tel VR::*hph*), 3 (N3440; TelVIIL::*bar*), 4 (N3456; TelIIR::*bar*) digested with the enzyme named at the top of each autoradiogram. Probes used are indicated under each panel and shown as gray bars in the maps.

As a first step to test for telomeric silencing in Neurospora, we inserted a selectable marker, *hph*, into a subtelomeric region by homologous recombination in strain N1982, see figure 2, which carries the *nst-1*^*RIP*1 ^mutation. The *hph *gene confers hygromycin resistance, even when *hph *is expressed at a low level. Our initial work relied on the two described Neurospora telomere sequences, IVL and VR [3,29]. From a screen of roughly 150 transformants by Southern hybridization, we identified a single clone with the correct integration near Tel VR; nearly all other transformants had ectopic insertions, as is typical in Neurospora. Several attempts to insert the selectable *mtr *allele into the subtelomeric region of telomere IVL failed to yield an isolate with a homologous integration.

**Figure 3:**
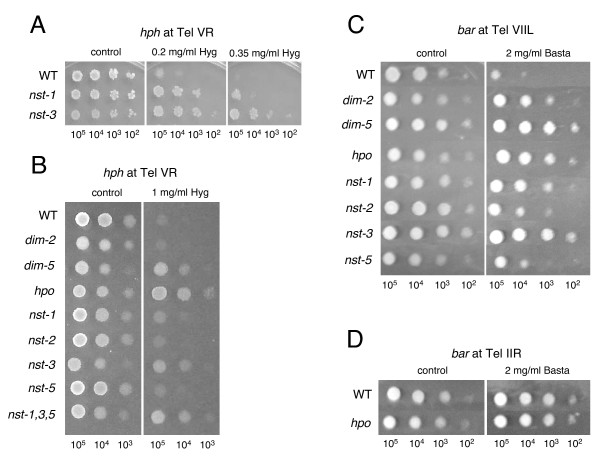
**Telomeric silencing depends on *dim-5*, *hpo *and *nst *genes.** Conidia from strains carrying the telomere VR *hph *allele were spotted at densities noted at the bottom of each panel to test sensitivity to moderate (A) or high (B) levels of Hygromycin. Strains used in A were: N2285 (*nst*^+^, "WT"); N2130 (*nst-1*^*RIP*1^); N2635 (*nst-3*^*RIP*1^). Strains used in B were: N3120 (*nst*^+^, "WT"); N2833 (*dim-2*); N2997 (*dim-5*); N3004 (*hpo*); N3125 (*nst-1*^*RIP*1^); N2667 (*nst-2*^*RIP*1^); N3126 (*nst-3*^*RIP*1^); N3130 (*nst-5*^*RIP*1^); N3132 (*nst-1, nst-3, nst-5*). C) Conidia from strains N3441 (*nst*^+^), N3447 (*dim-2*), N3449 (*dim-5*), N3443 (*hpo*), N3452 (*nst-1*^*RIP*1^), N3453 (*nst-2*^*RIP*1^), N3455 (*nst-3*^*RIP*1^), and N3445 (*nst-5*^*RIP*1^) were spotted on plates with or without Basta to assay expression of the *bar *transgene targeted to telomere VIIL. D) Conidia from strains N3457 (*nst*^+^) and N3456 (*hpo*) were spotted on plates with or without Basta to assay expression of the *bar *transgene targeted to telomere IIR.

We backcrossed the transformant with the desired insertion at Tel VR to an *nst-1*^+ ^strain and found that hygromycin resistance (Hyg^R^) was lost in progeny with the *nst-1*^+ ^allele, providing evidence for telomeric silencing in Neurospora and indicating that *nst-1 *plays a role in this process (Figure 3A and 3B). As a control, we inserted the *hph *construct at random ectopic chromosomal locations in a *nst-1*^+ ^strain by co-transformation with pBARKS1[39], which confers resistance to Basta. Strains bearing *hph *sequences were identified by Southern hybridization and were all found to be Hyg^R^, confirming that *hph *is expressed when not at a telomeric locus (data not shown).

To determine if more than one *nst *gene is required for telomeric silencing, we combined *nst *mutations described above with this *hph *marker and then tested the effect of the mutations on expression of *hph *at Tel VR. No loss of silencing was detected in spot tests with the *nst-2 *mutant (Figure 3B) but mutation of *nst-3 *resulted in striking derepression of *hph *(Figure 3A and 3B). Mutation of *nst-5 *also relieved silencing (Figure 3B). While the *nst-3 *mutant showed robust growth on hygromycin, the *nst-1 *and *nst-5 *mutants showed barely visible growth when 10,000 or fewer conidia were spotted. However, resistance to hygromycin was reproducibly greater than in a *nst*^+ ^strain, and growth was more robust at lower hygromycin concentrations (shown in Figure 3A for *nst-1*). As one approach to test the possibility that these genes are partially redundant, we constructed a strain defective for *nst-1, -3 *and *-5 *and compared its level of Hyg^R ^with those of the single mutants. The triple mutant showed a similar level of resistance to *nst-3 *(Figure 3B).

We also generated strains to test the possible effect on telomeric silencing of genes required for DNA methylation, namely *dim-2*, *dim-5 *and *hpo*, which respectively encode the DNMT responsible for all known methylation in Neurospora [23], the HKMT responsible of methylation of K9 on histone H3, and the adaptor protein HP1. Elimination of DNA methylation by mutation of *dim-2 *had no discernable effect on expression of *hph *at Tel VR (Figure 3B). In contrast, both the HKMT DIM-5 and HP1, which reads the mark created by DIM-5, were critical for silencing of this marker (Figure 3B).

### Silencing of *bar *at Telomere VIIL

To determine whether telomeric silencing occurs at other Neurospora telomeres and works on other genes, we inserted the selectable markers *bar*, encoding Basta resistance, and *ad-3A *proximal to two other telomeres, namely those of chromosome arms VIIL and IIR (Figure 2B and 2C, respectively). These novel telomere regions were identified by sequencing and mapping clones containing TTAGGG repeats (Wu C, Kim YS, Smith KM, Li W, Hood HM, Staben C, Selker EU, Sachs MS, Farman ML, unpublished). As the *hpo *mutation provided the strongest relief of silencing of *hph *at Tel VR, we used an *hpo *strain as the transformation host. To reduce ectopic integrations, the strain also included a mutation of *mus-52*, the gene that encodes the KU80 homolog required for non-homologous end-joining of DNA double strand breaks [40]. Transformation with the Tel VIIL targeting plasmid (pTTK19) yielded two Basta^R ^transformants and Southern hybridizations revealed that both had integrated the construct correctly (Figure 2B). Similarly, we obtained one Basta^R ^transformant with the Tel IIR-targeting plasmid pTTK22 and confirmed that this clone integrated the transgenes properly on LG IIR (Figure 2C). While use of *mus-52 *facilitated integration of markers into subtelomeric sites on VIIL and IIR, equivalent attempts to target to Tel IVL were unsuccessful.

The transformants with markers at VIIL and IIR were crossed to restore *hpo *function and test for silencing. We found that *bar *expression from the allele at Tel VIIL was lost in the *hpo*^+ ^background (Figure 3C). This implies that the telomeric silencing observed with *hph *at Tel VR was not a peculiarity of one chromosome or one marker. The *ad-3A *gene was expressed, even in the original transformant (data not shown) and we did not explore the possibility that its level of expression pattern depended on *hpo *or other markers. The Ku proteins play a role in telomere function in other organisms [41] so we tested the effect of the *mus-52 *mutation on expression of the Tel VIIL-targeted *bar *marker. The *mus-52 *mutation did not cause a loss of silencing (data not shown), as is also the case in *S. pombe *[42]. Interestingly, the Tel IIR *bar *allele was not silenced when introduced into an *hpo*^+ ^background (Figure 3D).

Having demonstrated telomeric silencing on two Neurospora chromosomes, we wished to test whether they showed a similar dependence on Sir2p homologues and other proteins implicated in heterochromatin formation. We therefore crossed strains bearing the marked Tel VIIL with strains bearing mutations in *dim-2, dim-5, hpo*, and the various *nst *genes that we found to be involved in telomeric silencing on chromosome V. Mutation of *dim-5, hpo, nst-1, nst-2, nst-3 *and *nst-5 *all caused loss of silencing of *bar *at Tel VIIL (Figure 3C), albeit to various extents. The results were generally consistent with the results obtained for these genes with *hph *at Tel VR but we were surprised to find an effect of the *nst-2 *mutation, as this gene had no noticeable effect on *hph *silencing at Tel VR. Another notable difference concerns the DNMT DIM-2. Unlike the situation for *hph *at Tel VR, the *dim-2 *mutation caused derepression of the *bar *marker at Tel VIIL (Figure 3C) suggesting differential involvement of DNA methylation at these telomeres.

### Inhibitors of HDACs relieve telomeric silencing

**Figure 4:**
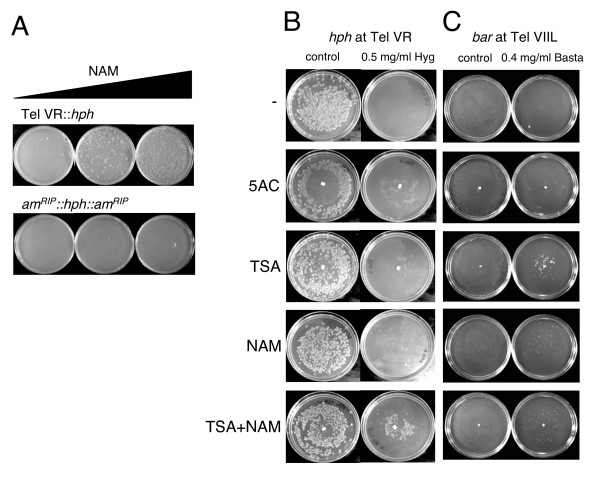
**Derepression of the subtelomeric transgenes by inhibitors of histone acetyltransferases and DNA methyltransferases.** A) Nicotinamide (NAM) relieved silencing of telomeric *hph *(N2285) but not *hph *flanked by *am*^*RIP *^(N2015) at a non-telomeric site. All plates contained 0.6 mg/ml Hygromycin. Plates with NAM contained 0.1 mg/ml (middle plate) or 1 mg/ml (right plate) of the drug. Each plate received approximately 100,000 conidia. B) Approximately 1000 conidia from an *nst*^+ ^strain (N3120) carrying the telVR *hph *allele were plated in the presence or absence of 0.5 mg/ml Hygromycin to assay the effect of the drugs 5-azacytidine (5AC), Trichostatin A (TSA) and NAM on telomeric silencing. C) Same as B except strain N3441 carrying the tel VIIL *bar *allele was plated in the presence and absence of 4 mg/ml Basta. Control plates lacking Basta show a lawn of growth.

The HDAC Class I (Rpd3-like) and II (Hda1-like) inhibitor trichostatin A (TSA) [43] was previously shown to cause selective inhibition of DNA methylation in Neurospora [44]. To further explore the mechanism of telomeric silencing in Neurospora, we tested the effect of inhibitors of DNA methylation and HDACs on the expression of *hph *at Tel VR and *bar *at Tel VIIL. We first used a plating assay to test the effect of the Class III (sirtuin) HDAC inhibitor nicotinamide [45] on TPE and also on expression of an interstitial allele of *hph *allele that was silenced by DNA methylation spreading from flanking sequences that had been subjected to RIP [46]. Nicotinamide caused relief of *hph *at Tel VR but not at the interstitial site (Figure 4A). This finding supports the genetic evidence that sirtuins are involved in telomeric silencing in Neurospora and implicates their HDAC activities. It also suggests that sirtuins are not required for maintenance of DNA methylation.

We adopted an assay involving a gradient of inhibitors for additional tests of the effects of nicotinamide, TSA and the DNA methylation inhibitor 5-azacytidine (5AC) on expression of the telomeric *hph *(Figure 4B) and *bar *(Figure 4C) alleles. Both nicotinamide and TSA allowed a small fraction of conidia to escape silencing and grow on hygromycin, suggesting that both NAD^+^-dependent and -independent HDACs play a role in telomeric silencing. Interestingly, repeated tests showed that when both drugs were added together, the loss of silencing was significantly greater for telomeric *hph*, implying overlapping functions of members from different HDAC classes. In contrast to transcriptional silencing caused by DNA methylation, silencing of *hph *at Tel VR was unaffected by the DNA methylation inhibitor 5AC. This is consistent with our findings with strains defective in DNA methylation due to mutations in the *dim-2 *gene (Figure 3B). A small number of colonies escaped silencing of *bar *on Tel VIIL in the presence of 5AC (Figure 4C), consistent with relief of *bar *silencing in a *dim-2 *mutant (Figure 3C). In summary, these data suggest that multiple HDACs are involved in telomeric silencing and that DNA methylation is not universally required for gene silencing in Neurospora.

### NST activity

**Figure 5:**
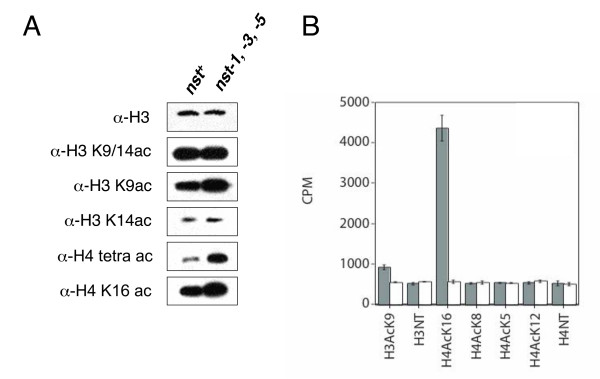
**Histone H4 acetylation is increased in *nst *mutants.** A) Western blots of nuclear extracts from strains N3120 (*nst*^+^) and N3132 (*nst-1*^*RIP*1^*nst-3*^*RIP*1^*nst-5*^*RIP*1^) probed for the indicated epitopes. Different exposure times were used (not indicated) to avoid saturation of signal. B)*In vitro *NAD^+^-dependent deacetylase assays with the indicated H3 or H4 peptide substrates (NT, unmodified N-terminal peptide). Grey bars show activity for purified NST-1 protein; white bars represent GST (only) control.

The *nst *genes were identified by homology to *SIR2*. Not all Sir2p homologues appear to be HDACs; some have been shown to have non-histone substrates or lack deacetylase activity toward all substrates tested (reviewed in [13]). In one approach to determine whether the NST proteins have HDAC activity, we used western blotting to assess whether mutation of *nst *genes affected the global level of histone acetylation at candidate residues. Initial tests revealed no change in the *nst-1*, *-3 *or *-5 *mutants (data not shown), consistent with the possibility that they are partially redundant. We therefore tested a triple (*nst-1 nst-3 nst-5*) mutant and, indeed, found significant hyperacetylation (Figure 5A) in contrast to the case with the single *nst *mutants. Although no change was detected with antibodies that recognize acetylated H3 K14 or acetylated H3 K9 and/or K14, an antibody against acetylated H3 K9 revealed increased acetylation in the triple *nst *mutant (Figure 5A). We also detected clearly increased acetylation of H4 in the *nst *triple mutant, both with H4 tetra-acetyl antibodies (recognizing acetyl K5, 8, 12, or 16) and with H4 K16-acetyl antibodies. These results are consistent with the idea that NST proteins are indeed HDACs.

**Figure 6:**
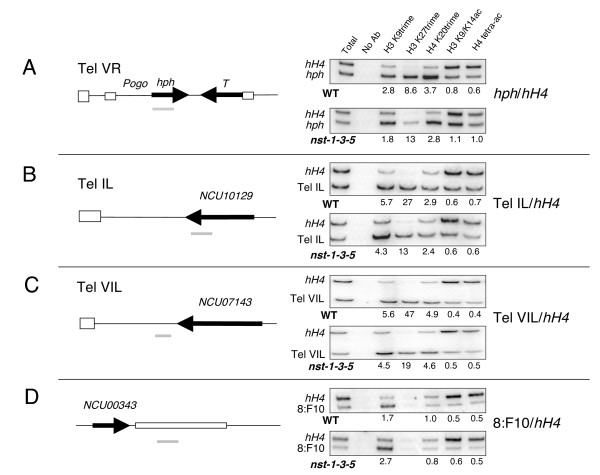
**Chromatin immunoprecipitation (ChIP) to compare histone modifications near telomeres in WT and *nst *triple mutant strains.** Telomeric regions investigated are shown in the schematics at left. Vertical striped bars represent telomeric (TTAGGG)_n _repeats and horizontal striped bars in A represent *Pogo *LTRs. Black bars represent open reading frames and are labeled according to the Broad Institute database. Strains N3120 (*nst-1*^+^) and N3132 (*nst-1*^*RIP*1^*nst-3*^*RIP*1^*nst-5*^*RIP*1^) show increased acetylation of H3 and H4 and decreased histone methylation at the derepressed *hph *gene at telomere VR. A-D) Immunoprecipitated chromatin was amplified with primers for the indicated regions, shown as gray bars. Values in the figure indicate enrichment of specific modifications at telomeric regions relative to euchromatic (hH4) regions (A-C). To determine these values bands were quantified using ImageQuant software and ratios were calculated relative to input DNA. D) We also tested a non-telomeric heterochromatic region with methylated DNA (8:F10) [27].

To directly test whether at least one of the predicted NST proteins is an HDAC, we expressed NST-1 in *Escherichia coli *and assayed it for NAD^+^-dependent deacetylase activity with various acetylated histone peptides. NST-1 displayed robust activity with an H4 peptide acetylated on K16 and weak activity with an H3 peptide acetylated on K9, but showed no activity on H4 peptides acetylated on K5, K8, or K12 (Figure 5B). Thus, the *in vitro *assays support the *in vivo *findings (Figure 5A and Figure 6, below) and indicate that at least one NST protein is a bona fide NAD^+^-dependent histone deacetylase.

### DNA methylation of subtelomeric regions

**Figure 7:**
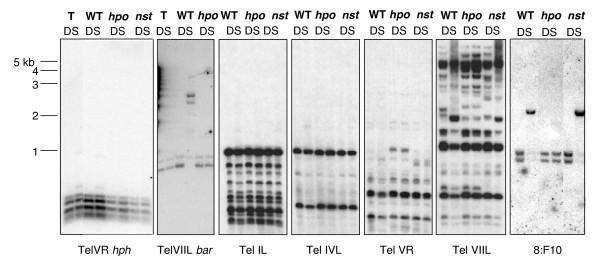
**Analyses of DNA methylation near telomeres.** DNA was digested with isoschizomers DpnII (D) and Sau3AI (S), separated on agarose gels, blotted, and probed with telomere proximal regions to assay DNA methylation in these regions. Strains for the TelVR *hph *panel: N2292, the primary transformant with *hph *targeted to tel VR (T); N3120, a *nst*^+ ^strain (WT); N3004, an *hpo *strain (*hpo*); N3132, the triple *nst-1, -3, -5 *mutant (*nst*). The *hph *probe was the first 600 bp of the coding region. Strains for the tel VIIL *bar *panel: N3440, the primary transformant with *bar *targeted to tel VIIL (T); N3441 (*nst*^+^); N3443 (*hpo*). The *bar *probe was the entire coding region. For the other panels, DNA samples of a wildtype strain, N150 (WT), an *hpo *mutant, N3004 (*hpo*), and N3132, a triple *nst-1 nst-3 nst-5 *mutant (*nst*) were processed as for the other panels and probed for: telomere IL (Tel IL; 226–1249 bp from end); telomere IVL (TelIVL; 2500–3657 bp from end); telomere VR (Tel VR; 1130–2887 bp from end); telomere VIIL (Tel VIIL; 324–1306 bp from end); a methylated repeat called 8:F10 (see text).

Most available information on the structure and sequence of telomeres and subtelomeric heterochromatic regions is from organisms that do not have DNA methylation. Thus we were interested to examine both native and introduced sequences associated with telomeres for methylation in Neurospora. We found evidence of light DNA methylation at the silenced Tel VR *hph *marker and robust DNA methylation at the Tel VIIL *bar *marker (Figure 7). This is consistent with our observation that *dim-2 *caused derepression of the *bar *marker at VIIL but did not affect *hph *expression at VR (Figure 3B and 3C). We also detected limited methylation at some, but not all, unmarked telomeres. Specifically, *Dpn*II/*Sau*3AI sites were lightly methylated or unmethylated at telomeres IL, IVL, and VR but substantially methylated at Tel VIIL (Figure 7).

To examine whether the telomeric methylation is dependent on HP1, as found for DNA methylation at other described chromosomal sites [26], we tested the effect of a null mutation of *hpo. *In addition, we tested the effect of mutating the three *nst *genes that we had found resulted in hyperacetylation of histones H3 and H4 (Figure 5). Introduction of the *hpo *mutation introduced a restriction fragment length polymorphism (RFLP) (Tel VR) but it clearly also resulted in loss of methylation at all regions tested, consistent with previous indications that *hpo *causes a global loss of methylation [26]. In contrast, DNA methylation of subtelomeric regions was essentially unaffected in the triple *nst *mutant. We also tested the effect of the *nst *mutation on methylation of a non-telomeric repeated element (8:F10) [27] and found no effect on DNA methylation in this region (Figure 7).

### Histone modifications associated with silent telomeric regions

Little information is available about histone modifications in telomeric regions and no such information was available for Neurospora. We therefore used chromatin immunoprecipitation (ChIP) to characterize key modifications of histones H3 and H4 associated with sequences near Neurospora telomeres. Both marked and unmarked telomeres were examined because of the possibility that introduced markers could influence the epigenetic state of the chromatin. Results from our genetic studies with *dim-5*, *hpo *and *nst *mutants and from western blots suggested that the TAS would at least sport methylated K9 on H3 and would perhaps also show hypoacetylated histones. We found reproducible differences between telomeric regions and interstitial euchromatic regions (for example, histone H4 gene, *hH4*) and, interestingly, we also found differences between the telomeric regions and interstitial heterochromatic regions, that is, regions that had undergone RIP and whose DNA is methylated (8:F10). Like previously tested products of RIP bearing DNA methylation, the marked (*hph; *Figure 6A) and unmarked (Tel IL & Tel VIL; Figure 6B and 6C, respectively) telomere-associated regions showed hypermethylation of H3 K9. Unlike interstitial heterochromatic sequences (Figure 6D and Honda S, Lewis Z, Selker EU, unpublished), however, the telomere-associated regions showed a striking enrichment for trimethylated H3 K27 and trimethylated H4 K20. In addition, both the marked and unmarked telomeric sequences showed hypoacetylation relative to the control euchromatic sequence (*hH4*).

The triple *nst *mutant attenuated the hypermethylation of H4 K20 and hypoacetylation of H3 and H4 at the marked telomere (note the ratio of telomeric to euchromatic PCR product normalized to the input DNA sample in Figure 6A). Whereas in the wild type the anti-histone H3 K9/K14 antibody produced a relative enrichment (*hph/hH4*) of 0.8 and the anti-tetracetylated histone H4 antibody produced a relative enrichment of 0.6 (Figure 6A), both H3 (K9/14) and H4 acetylation levels were equivalent for *hph *and *hH4 *in the *nst *triple mutant; thus, loss of silencing in the triple *nst *mutant correlated with a level of histone acetylation at telomeric *hph *typical of euchromatin. These results are consistent with the western blot results showing increased acetylation on both H3 and H4 in the *nst *triple mutant (Figure 5A). Equivalent results were found for the 5' end of the *hph *gene (shown in Figure 6A) and the middle of the coding region (data not shown). Although the calculated enrichment of *hph*/*hH4 *for H3 K27 trimethylation went up from 8.8 in wild type to 13.3 in the mutant, the intensity of the PCR product is greatly reduced in the mutant, which suggests that H3 K27 trimethylation of *hph *is also reduced in the triple *nst *mutant. Although H3 trimethyl-K9, H3 trimethyl-K27, and H4 trimethyl-K20 were enriched at the unmarked subtelomeric sequences, as for the telomeric *hph*, these modifications appeared mostly unchanged in the triple *nst *mutant. Only H3 trimethyl-K27 showed a reproducible change; it decreased about two-fold in the mutant (Figure 6B and 6C). The *nst *mutations did not effect DNA methylation at non-telomeric heterochromatic sequences (Figure 7, 8:F10) and also did not appear to greatly affect histone modifications at the region examined (8:F10; Figure 6D).

**Figure 8:**
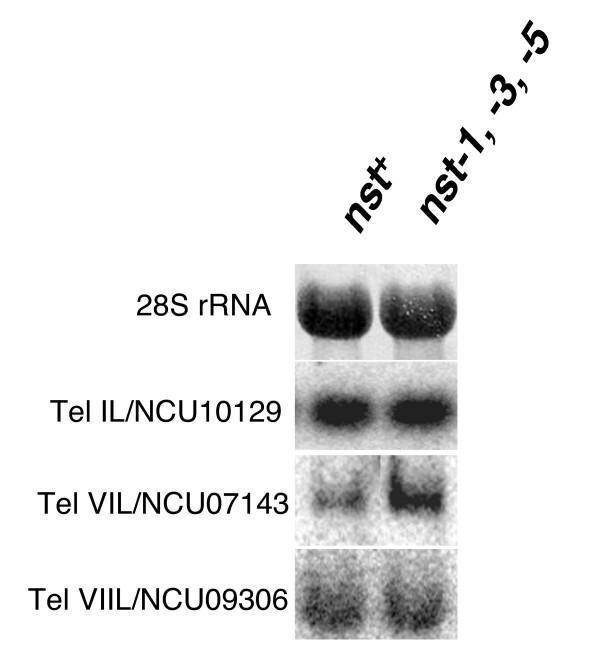
**Expression of genes near telomeres is unaffected by *nst *mutations.** Total RNA was extracted [47] from N3120 (*nst*^+^) and N3132 (*nst-1*^*RIP*1^*nst-3*^*RIP*1^*nst-5*^*RIP*1^) and 20 μg was separated by agarose gel, blotted, and probed as described [48]. The 28S rRNA panel shows equivalent loading in both lanes by methylene blue staining of a blot. Other panels are autoradiograms after probing with the coding region of the gene closest to telomere IL (NCU010129.3), VIL (NCU07143.3), or VIIL (NCU09306.3).

We also examined native Tel VIIL at the site where the *bar *gene was silenced and found comparable patterns of increased/decreased histone modifications, relative to control euchromatic sequences (data not shown). Interestingly, we also found hypoacetylation of H3 and H4 at all subtelomeric regions examined, including actively transcribed genes. The Tel IL and VIL primers amplify genes NCU10129.3 and NCU07143.3, respectively, both of which we found to be active genes. Northern analysis with RNA isolated from wild type and the triple *nst *strains showed that the expression of these genes, like their histone acetylation status, was comparable in the wild type and the triple *nst *mutant (Figure 8) [47,48]. In summary, changes in histone modifications in the *nst *triple mutant are localized to the Tel VR-targeted *hph *allele, with the exception of a general effect of reduction of H3 trimethyl-K27.

## Discussion

### Structural similarities and differences between telomeres of Neurospora and other model organisms

The structure of the currently known Neurospora telomere sequences [49] is quite simple. The length of Neurospora (TTAGGG)_n _repeats (~200 bp) is roughly ten-fold less than in mammals [29]. Unlike other fungi and mammals (reviewed in [50]), subtelomeric regions in Neurospora do not consist of tandem repeats and do not contain a particular class of repeat element present at each telomere, or even at a subset of the characterized telomeres. Rather, a short stretch of AT-rich DNA (roughly 2 kb long but variable in length) separates the first telomere repeat unit from the most telomere-proximal gene (Wu C, Kim YS, Smith KM, Li W, Hood HM, Staben C, Selker EU, Sachs MS, Farman ML, unpublished). The evolutionary origin of this telomere arrangement likely reflects the action of a genome defense system called RIP in Neurospora [38]. The RIP machinery detects duplicated sequences and changes C:G to T:A base pairs, presumably by deamination of cytosines or methylcytosines [51]. The resulting AT-rich sequences serve as targets for the DNA methyltransferase DIM-2 [23], which typically methylates remaining cytosines [52-54]. Subtelomeric sequences of Neurospora show hallmarks of RIP, namely a deficiency of CpA dinucleotides and a corresponding overabundance of TpA dinucleotides, the RIP machinery's preferred substrate and product, respectively [52], and DNA methylation (Tel VIIL; Figure 7). The current subtelomeric sequences may have once shared homology that is now unrecognizable because of RIP and evolutionary divergence.

### Telomeric markers for Neurospora

The simple structure of Neurospora subtelomeres facilitated our strategy to insert selectable markers near Neurospora telomeres. For Tel VR, we chose to insert a copy of *hph *within a *Pogo *transposon relic found directly adjacent to the telomere repeat (Figure 2A). We also inserted the *T *gene (encoding tyrosinase) at Tel VR, and expression of this allele of *T *correlated with expression of *hph *in *nst-1 *and *nst-3 *strains (data not shown). We did not analyze expression of this allele further as RIP may have been induced by the native copy of *T *in the Neurospora genome during successive crosses. For Tel VIIL, the *bar *and *ad-3A *markers were inserted within the coding region of an expressed gene, NCU09306.3, which is directly flanked by an unnamed RIP-mutated repeated element found at more than ten other chromosomal positions (none of which is within 100 kb of a known telomere) and a CenVII-like RIP-mutated region, which directly flanks the (TTAGGG)_n _telomere repeats (Figure 2). Thus, it is likely that NCU09306.3 is embedded in heterochromatin even though it is expressed, as is also the case for NCU10129.3, at Tel IL (Figure 6B and 8). ChIP data supported this possibility, showing an enrichment of histone methylation and absence of histone acetylation at the 3'end of NCU09306.3 (data not shown). Most importantly, our genetic data show that a marker inserted in NCU09306.3, like one inserted into a non-genic region near another telomere, is subject to telomeric silencing involving both histone deacetylation and histone methylation.

### Involvement of histone deacetylases in telomeric silencing

The first evidence for telomeric silencing in Neurospora came from our observation of increased expression of the *hph *gene in a strain in which we had mutated *nst-1*, the closest homologue of *S. cerevisiae SIR2. *We then found evidence of extensive involvement of other deacetylases. The role of protein deacetylases, such as HDACs, is complex because of their multiple and partially overlapping functions. In *S. pombe*, telomeric silencing is dependent on deacetylation of histone H3 K9 and H4 K16 by Sir2, and subsequent methylation of histone H3 K9 [15,16]. A Sir2 deletion strain showed increased H3 K9 and H3 K14 acetylation at a subtelomeric region, and reduced H3 K9 methylation and Swi6/HP1 binding. Mutation of the Class I and II HDACs (Clr6 and Clr3, respectively) or treatment with the Class I/II HDAC inhibitor TSA also caused increased expression of telomere-linked genes [55,56]. In human cells, TSA, but not the sirtuin inhibitor sirtinol, caused derepression of a subtelomeric reporter and mislocalization of HP1 [57]. A recent study, however, showed SIRT6 is an H3 K9 deactylase that localizes to telomeres and is required for telomere integrity [58]. Little or no effect on subtelomeric gene expression was observed in mouse embryonic stem cells treated with TSA [59]. As such negative results of drug treatment assays are difficult to interpret, it remains possible that both Class I/II and III HDACs are required for mammalian telomere silencing; additional genetic studies may shed light on this.

Our finding that NST-1 is a NAD-dependent (Class III) HDAC, as expected, fits our observation that nicotinamide is a potent inhibitor of telomeric silencing in Neurospora (Figure 4). The finding that TSA also interfered with silencing strongly suggests that Class I or Class II HDACs are also important. Thus, it should be interesting to further explore the importance of both NAD-dependent and -independent HDACs in telomeric silencing. Increased acetylation at H3 K9 would of course be expected to interfere with methylation of this residue, but various HDACs may also be involved in silencing at other levels. For instance, *N. crassa *NST-1, *S. pombe *Sir2, *S. cerevisiae *Sir2p, and human HST-1 all appear to target H4 K16 for deacetylation. In the absence of these proteins, increased H4 K16 acetylation may somehow interfere with silencing. Our observation that loss of telomeric silencing was enhanced when nicotinamide and TSA were added in combination suggests overlapping roles of HDACs of Class I/II and Class III. It should be interesting to test mutants in the four Neurospora Class I and II HDACs (*hda-1 *through *hda-4*) [31] for their involvement in telomere silencing.

The finding that a triple *nst *mutant showed a greater increase in histone acetylation, as assayed by western blot of nuclear proteins and ChIP (Figures 5A and 7 and data not shown), than the individual *nst-1, -3*, or *-5 *mutants suggests that two or more of the *nst *genes have partially overlapping functions. The *nst-2 *mutant had no discernable effect on silencing of the *hph *at Tel VR, but reduced silencing of *bar *at Tel VIIL. Curiously, its closest homologues studied, *S. cerevisiae *Hst2 [36] and human SIRT2 [32], are cytosolic. Altogether, available information from Neurospora and other organisms is most consistent with all three classes of HDACs playing a role in telomeric silencing. It is also important to note that while individual mutants of *nst-1*, *nst-3*, or *nst-5 *caused a partial loss of telomeric silencing, there was no increase in histone acetylation assayed by western blot or ChIP in these mutants. It is possible that one or more of these genes encodes a protein that causes an indirect effect on telomere silencing.

### Trimethyl-K9 in histone H3 and HP1 are required for telomeric silencing in Neurospora

We found that methylation of histone H3 K9, a mark produced by DIM-5 and required for DNA methylation [24,25], is required for telomere silencing (Figure 3B and 3C) and enriched at all telomeres tested (Figure 6). Similarly, HP1, which binds methylated H3 K9 and is thought to be essential for heterochromatin formation [26], is required for telomere silencing (Figure 3B and 3C). ChIP experiments showed that the silent *hph *transgene is marked with H3 K9 trimethylation as well as trimethyl H3 K27 and H4 K20 (Figure 6). This is the first report of a role for H3 K27 methylation in Neurospora, and an important observation that shows Neurospora telomeres better model mammalian telomeres than those of the yeasts, which completely lack H3 K27 methylation (and DNA methylation). All three histone methylation marks were modestly reduced in an *nst-1, -3, -5 *triple mutant, with the most striking reduction in H3 K27 methylation. Once the histone methyltransferases (HKMTs) responsible for methylation of H3 K27 and H4 K20 methylation are identified, it will be interesting to test whether these marks, like H3 K9 methylation, are required for telomeric silencing. H3 K27 methylation was not observed in other regions of heterochromatin (for example, 8:F10) so we hypothesize it is involved in telomere silencing but not DNA methylation.

### DNA methylation is not generally required for silencing at Neurospora telomeres

Mutation of the *dim-2 *gene, which is responsible for all known DNA methylation in Neurospora [23], did not cause loss of silencing of the *hph *gene targeted to Tel VR (Figure 3B). Similarly, treatment with the DNA methylation inhibitor 5AC did not relieve silencing (Figure 4B). We conclude that the light DNA methylation observed at telomere VR (Figure 7) is not required for silencing in this region. This is consistent with the observation that 5AC had no effect on silencing of a telomeric transgene in human cancer cell lines [57]. Interestingly, telomeric transgenes in mouse embryonic stem cell lines become increasingly silenced and methylated as they are passaged and the eventual tight silencing and DNA methylation are reversible by 5AC [59].

In contrast to the situation at Tel VR, the sequences immediately proximal to Tel VIIL were significantly methylated (Figure 7) and DNA methylation was responsible for some silencing of *bar *inserted in this region. Mutation of *dim-2 *or treatment with 5AC both relieved silencing (Figures 3 and 4). It is interesting that *dim-5 *and *hpo *are required for telomeric silencing in Neurospora but DNA methylation, although frequently present, is not universally required to maintain silencing. Our findings provide the first examples of H3 K9 methylation acting to silence a gene independently of DNA methylation in Neurospora. It is noteworthy that mammals also use common factors for DNA methylation and telomere silencing [1] and telomere length is regulated by H3 K9 specific HKMTs in mouse [60]. Shortening of telomeres associated with aging is associated with changes in chromatin structure that alter the expression of neighboring genes [61].

In summary, our study on the structure and expression of sequences at Neurospora telomeres demonstrates the existence of telomeric silencing in Neurospora. We show important similarities, but also differences, in the silencing of markers integrated at subtelomeric regions. We also showed that at least one member of the Neurospora sirtuin family of NAD-dependent HDACs, NST-1, is a bona fide H4 K16 HDAC. Importantly, our work reveals an additional role for H3 K9 methylation and HP1 in Neurospora, independent of DNA methylation. It also provides evidence for the involvement of multiple classes of HDACs, including members of the NAD-dependent and -independent classes, and H3 K27 and H4 K20 HMTs. One major question remaining is how exactly heterochromatin is nucleated at Neurospora subtelomeres, as the Neurospora genome contains no good homologues of *S. pombe *Taz1 or mammalian telomere repeat binding factors.

## Methods

### Neurospora strains and growth conditions

A list of *N. crassa *strains used in this study is provided in Table 1[62,63]. Standard conditions were used for their growth and maintenance [64].

**Table 1:** List of strains

**Strain Number**	**genotype**	**reference**
N150	*mat A*	FGSC2489

N534	*mat a; mtr*^*SR*62^*col-4; trp-2*	[62]

N564	*mat A; Δmtr::hph*	this study

N565	*mat A; mtr*^*SR*62^*col-4; trp-2*	this study

N593	*mat a; arg-12*	this study

N1275	*mat a; dim-2 arg-10*	[23]

N1445	*mat a his-3; am*^132^*inl*	this study

N1447	*mat a his-3; inl*	this study

N1674	*mat A his-3; lys-1 am*^132^*inl; am*^*RIP*^::*hph::am*^*RIP*^	[63]

N1877	*mat his-3; Δdim-2::hph*	[23]

N1982	*mat A; mtr col-4; nst-1*^*RIP*1^*trp-2*	this study

N2015	*his-3 cyh-1; am*^132^*inl; am*^*RIP*^::*hph::am*^*RIP*^	this study

N2130	*mat a; mtr col-4; telVR::hph::T; nst-1*^*RIP*1^*trp-2*	this study

N2140	*mat A; dim-5 leu-2 pan-1*	[25]

N2225	*nst-1*^*RIP*1^; *am::hph::am*	this study

N2264	*mat a his-3; dim-5 leu-2 pan-1*	[25]

N2284	*mat A; mtr col-4; telVR::hph::T; nst-1*^*RIP*1^*trp-2*	this study

N2285	*mat A; mtr col-4; telVR::hph::T; trp-2*	this study

N2288	*mat a; mtr col-4; telVR::hph::T; nst-1*^*RIP*1^*trp-2*	this study

N2292*	*mat A; mtr col-4; telVR::hph::T; nst-1*^*RIP*1^*trp-2*	this study

N2552	*mat A his-3*^+^::*hpo*^*RIP*^::*gfp; hpo*^*RIP*2^	[26]

N2633	*mat a nst-2*^*RIP*1^; *inl*	this study

N2634	*mat A nst-3*^*RIP*1^; *am*^132^*inl; am*^*RIP*^::*hph::am*^*RIP*^	this study

N2635	*mat A nst-3*^*RIP*1 ^*his-3*^+^::*nst-3*^*RIP*^;*mtr col-4; telVR::hph::T; trp-2*	this study

N2636	*mat A nst-3*^*RIP*1^; *mtr col-4; telVR::hph::T; nst-1*^*RIP*1^*trp-2*	this study

N2664	*mat a; mtr col-4; telVR::hph::T*	this study

N2667	*mat a nst-2*^*RIP*1^; *telVR::hph::T*	this study

N2681	*mat A his-3*^+^::*nst-5*^*RIP*2^; *nst-5*^*RIP*1^; *am*^*RIP*^::*hph::am*^*RIP*^	this study

N2693	*mat a; nst-5*^*RIP*1^; *telVR::hph::T*	this study

N2833	*mat A; telVR::hph::T; dim-2 arg-10*	this study

N2921	*mat a nst-3*^*RIP*1^; *mtr col-4; lys-1 inl; trp-2; am*^*RIP*^::*hph::am*^*RIP*^	this study

N2997	*mat A; dim-5 leu-2 pan-1; telVR::hph::T*	this study

N3004	*mat A; telVR::hph::T; hpo*^*RIP*2^	this study

N3017	*mat A Δad-3A; Δmus-52::hph; hpo*^*RIP*2^*trp-2*	this study

N3120	*mat a; telVR::hph::T*	this study

N3125	*mat a; telVR::hph::T; nst-1*^*RIP*1^	this study

N3126	*mat A nst-3*^*RIP*1^; *telVR::hph::T; trp-2*	this study

N3130	*mat a; nst-5*^*RIP*1^; *telVR::hph::T; trp-2*	this study

N3132	*mat a nst-3*^*RIP*1^; *nst-5*^*RIP*1^; *telVR::hph::T; nst-1*^*RIP*1^	this study

N3440*	*mat A Δad-3A; Δmus-52::hph; hpo*^*RIP*2^*trp-2; telVIIL::ad-3A*^+^::*bar*	this study

N3441	*mat a; telVR::hph::T*; *telVIIL::ad-3A*^+^::*bar*	this study

N3442	*mat a; Δmus-52::hph*;*telVR::hph::T*; *telVIIL::ad-3A*^+^::*bar*	this study

N3443	*mat A; hpo*^*RIP*2^; *telVIIL::ad-3A*^+^::*bar*	this study

N3445	*mat A Δad-3A; nst-5*^*RIP*1^; *telVIIL::ad-3A*^+^::*bar*	this study

N3447	*mat A Δad-3A; telVIIL::ad-3A*^+^::*bar Δdim-2::hph*	this study

N3449	*mat A; dim-5 leu-2 pan-1; telVIIL::ad-3A*^+^::*bar*	this study

N3452	*mat a Δad-3A; nst-1*^*RIP*1^*trp-2; telVIIL::ad-3A*^+^::*bar*	this study

N3453	*mat a nst-2*^*RIP*1^; *trp-2; telVIIL::ad-3A*^+^::*bar*	this study

N3455	*mat a nst-3*^*RIP*1^; *trp-2; telVIIL::ad-3A*^+^::*bar*	this study

N3456*	*mat A Δad-3A; telIIR::ad-3A*^+^::*bar; Δmus-52::hph; hpo*^*RIP*2^*trp-2*	this study

N3457	*mat a; telIIR::ad-3A*^+^::*bar*	this study

N3459	*mat a; telIIR::ad-3A*^+^::*bar; Δmus-52::hph*	this study

N3460	*mat A; telIIR::ad-3A*^+^::*bar*; Δ*dim-2::hph*	this study

N3462	*mat A; telIIR::ad-3A*^+^::*bar; dim-5 leu-2 pan-1*	this study

N3458	*mat A; telIIR::ad-3A*^+^::*bar; nst-1*^*RIP*1^	this study

N3464	*mat a nst-3*^*RIP*1^; *telIIR::ad-3A*^+^::*bar*	this study

N3465	*mat A; telIIR::ad-3A*^+^::*bar; nst-5*^*RIP*1^	this study

*primary heterokaryotic transformant

### Protein sequences

Accession numbers for protein sequences in Figure 1 are as follows: *S. cerevisiae *Sir2p [GenBank:NP_010242]; *N. crassa *[GenBank:NST-1 XP_960372], NST-2 [GenBank:XP_963725], NST-3 [GenBank:XP_963711], NST-4 [GenBank:XP_959116], NST5 [GenBank:XP_956588], NST6 NCU05973.1 (this gene is misannotated in the current Broad database assembly) [49], NST7 [GenBank:XP_962799]; *H. sapiens *SIRT1 [GenBank:NP_036370], SIRT2 [GenBank:AAK51133], SIRT3 [GenBank:NP_036371], SIRT4 [GenBank:NP_036372], SIRT5 [GenBank:NP_036373], SIRT6 [GenBank:AAH28220], SIRT7 [GenBank:NP_057622].

### Mutagenesis by RIP

The *nst-1 *gene was amplified from genomic DNA with primers nst-1-1 and nst-1-2 (all primer sequences are listed in Table 2). The BamHI+XbaI-digested PCR product was cloned into the BamHI+XbaI-digested *his-3 *targeting vector pBM61 [65], generating pBM61-Sir2. pBM61-Sir2 was linearized with *Nde*I and targeted to the *his-3 *locus in strain N1674 by electroporation [65]. A *his-3*^+^::*nst-1 *transformant was crossed to strain N593 and random progeny was analyzed for evidence of RIP by Southern blotting. The endogenous *nst-1 *locus from one strain (N2225) showed RsaI RFLPs and was sequenced to identify the RIP mutations.

**Table 2:** Primer sequences

**Name**	**sequence**
nst-1-1	GCGGATCCGGTACAAACGGGCCGTTCTG

nst-1-2	GTTCTAGACCTAACGAACCTAGCCGGACC

S2L1-EcoRI	TCGAATTCCAAGGCGAAACATCACGCTTATTCT

S2L1-SpeI	TGACTAGTTGATCACAGCCACCGAGATCGTCTG

S2L2-EcoRI	CAGAATTCATGGACTGCTTGCGACCGAAACCGTCCG

S2L2-NotI	CCGCGGCCGCGTCGATCAGATGCCCTATACCCCGAG

203-4	GGCGGATCCATGTCCCTAGCGACAACA

203-5	CCTTAATTAAGCGGACCCGTCCAGTAAACAA

ad-3AF	GCCGCGGCCGCAGTCAAATGGAAGACGGTGG

ad-3AR	GCCGGATCCTCAAAGCTCACCAAGGGC

Tel7L5FAatII	GCCGACGTCATTATAGGACGAAAAGGG

Tel7L5RPmlI	GCCCACGTGTTGACATAGGCACTTGCC

Tel7L3FBamHI	GCCGGATCCCGAGTCAGCAAGAAGTTTTG

Tel7L3REcoRI	GCCGAATTCGGTTGAGCGGGTAGTTCC

Tel2R5FAatII	GCCGACGTCAGATGCTTATACTTAGGG

Tel2R5RPmlI	GCCCACGTGCAAAAAGCAATAGGAGGG

Tel2R3FBamHI	GCCGGATCCCGTTATTCTAAAGGACCC

Tel2R3RecoRI	GCCGAATTCCTAGTATTGAAAGGGGAG

nst1BamHI	GCGGATCCCTGAAGCTTGCACTCCGGAGGAAGCGGTG

nst1NotI	CTGCGGCCGCGAATGAGTTGTGAAATACCCGATCCAAACC

hH4-1F	AACCACCGAAACCGTAGAGGGTAC

hH4-1R	ATCGCCGACACCGTGTGTTGTAAC

8:F10F	GTAACGCAAATTCTAAAATTGCAATAC

8:F10R	CTTAGTAATTAATTTAATACGTGCGCC

hphF	GACCCGGTCATACCTTCT

hphR	TTCCCCAATGTCAAGCAC

telILF	CTTCTTGCGTCTTGCCTGCTC

telILR	CCTTTTCGTTCGGTTGACAGC

telVILF	AACTTGGCACCCTCCGCGTT

telVILR	CCCCTCTAAGTTTTCCGATT

The *nst-2 *gene was amplified from genomic DNA with primers S2L1-EcoRI and S2L1-SpeI, digested with EcoRI+SpeI, and cloned into EcoRI+SpeI-digested pBM61. The resulting plasmid, pBM61-S2L1, was linearized with NdeI and targeted to *his-3 *in strains N1445 and N1674. To mutate *nst-3*, the gene was amplified with primers S2L2-EcoRI and S2L2-Not1, digested with EcoRI+NotI, and cloned into EcoRI+NotI-digested pBM61. The resulting plasmid, pBM61-S2L2, was linearized with NdeI and targeted *his-3 *in strains N1445 and N1674. For both *nst-2 *and *nst-3*, His^+ ^transformants of N1445 and N1674 were crossed to each other and progeny were analyzed for evidence of RIP by Southern blotting of RsaI-digested DNA; RIP mutated alleles were then sequenced.

The *nst-5 *gene was amplified with primers 203-4 and 203-5, digested with BamHI+PacI and inserted into BamHI+PacI-digested pMF272 [66]. The resulting plasmid, pKA13, was linearized with DraI and targeted to *his-3 *in strain N1674. A His^+ ^transformant was crossed to strain N1447 and two progeny with duplications were then crossed to induce RIP. Southern analysis revealed RFLPs in strain N2681; thus the endogenous allele was sequenced.

### Insertion of markers at telomeres

The bacterial *hph *gene under the control of the constitutive *trpC *promoter was removed from pCSN43 [67] by digestion with SalI and ligated to XhoI-digested pGRG-1/TYR103 [68] to generate pCM8. To generate pCM11, pCM8 was digested with PvuII and the *hph *fragment was ligated to HpaI-digested pNC36 [3], which contains the *Pogo *transposon from the subtelomere of linkage group VR. BsiWI-digested pCM11 was used to transform strain N1982 (derived from a cross between strains N2225 and N565, which was derived from a cross between strains N564 and N534). Correct integration at LG VR was confirmed by Southern blotting in the transformant, N2292, and this strain was backcrossed to strain N534 and WT (N2285). We identified *nst-1*^*RIP*1 ^progeny that retained *telVR::hph::T *for further study (strains N2284, N2288). A Southern blot demonstrating subtelomeric integration is shown in Figure 3. The *nst-3*^*RIP*1 ^allele was then introduced by crossing N2284 and N2634 to obtain strains N2635 and N2636. Strains N2288 and N2634 were also crossed to generate N2664, which was crossed to strains N1275 (*dim-2*), N2140 (*dim-5*), N2552 (*hpo*) and N2681 (*nst-5*) to introduce the designated mutation (yielding strains N2833, N2997, N3004, and 2693, respectively). N2633 (*nst-2*) was crossed to N2284 to generate N2667. N2636 was crossed to N2693 to generate the triple *nst *mutant, N3132. A *nst*^+ ^strain (N3120) and *nst-1*^*RIP*1 ^(N3125), *nst-3*^*RIP*1 ^(N3132) and *nst-5*^*RIP*1 ^(N3130) siblings from this cross were also selected for further study. Complete genotypes are listed in Table 1.

The *ad-3A *coding region with its promoter was amplified from genomic DNA with primers ad-3AF and ad-3AR. To generate pTTK17, the *ad-3A *PCR product was digested with NotI+BamHI and ligated to pBARKS1 [69] digested with the same enzymes. Plasmid pBARKS1 contains the *bar *gene under the control of the *trpC *promoter, which allows selection for glufosinate ('Basta') resistance. A region near Tel VIIL (324–1306 bp from the chromosome end) was amplified by PCR with primers Tel7L5FAatII and Tel7L5RPmlI, digested with PmlI+AatII and ligated to pTTK17 DNA that had been digested with the same enzymes, yielding plasmid pTTK18. A second region of Tel VIIL (1310–2319 bp from the chromosome end) was amplified with primers Tel7L3FBamHI and Tel7L3REcoRI, digested with BamHI+EcoRI and ligated to pTTK18 digested with the same enzymes, yielding the gene targeting vector pTTK19. AlwNI-linearized pTTK19 was transformed into N3017 and Basta^R ^colonies were selected. Following confirmation of correct insertion by PCR analysis and Southern blotting (Figure 3), one transformant (N3440) was crossed to strains N2130 (*nst-1*), N2633 (*nst-2*), N2921 (*nst-3*), N2693 (*nst-5*), N1877 (*dim-2*), and N2264 (*dim-5*). Progeny that retained the *bar *marker at Tel VIIL but not the Δ*mus52::hph*^+ ^allele, and that also included the desired *nst *and *dim *mutations, were selected for further studies.

The same *ad-3A bar *construct was also targeted to the subtelomeric region of Tel IIR. A region near Tel IIR (1444–2472 bp from the chromosome end) was amplified with primers Tel2R3FBam and Tel2R3REco, digested with BamHI+EcoRI and ligated to pBARKS1 [69] digested with the same enzymes, yielding plasmid pTTK20. The *ad-3A *region was amplified and inserted into pTTK20 as described above, yielding pTTK21. A second region of Tel IIR (259–1278 bp from the chromosome end) was amplified with primers Tel2R5FAatII and Tel2R5RPmlI, digested with AatII+PmlI and ligated to pTTK21 DNA digested with the same enzymes, yielding gene targeting vector pTTK22. AlwNI-linearized pTTK22 was transformed into N3017 and Basta^R ^colonies were selected. Following confirmation of correct insertion by PCR analysis and Southern blotting (Figure 3), one transformant (N3456) was crossed to strains N2130 (*nst-1*), N2633 (*nst-2*), N2921 (*nst-3*), N2693 (*nst-5*), N1275 (*dim-2*), and N2264 (*dim-5*). As above, progeny that retained the *bar *marker at Tel IIR but not the Δ*mus52::hph*^+ ^allele, and that also carried the desired *nst *and *dim *mutations were selected for further studies.

### Telomeric silencing assay

Conidia from strain N3120 (telVR::*hph*^+^) or N3441 (tel VIIL::*bar*^+^) were collected in water from flasks containing Vogel's minimal medium, 2% sucrose, 1.5% agar and appropriate supplements after 1 week of growth at 32°C. Roughly 1000 conidia were spread on Vogel's minimal agar plates with 2% sorbose, 0.05% fructose and 0.05% glucose (FGS) and allowed to dry briefly. Nicotinamide was included in agar medium at 1 mg/ml. For the other drugs, 2 mm × 2 mm filter paper squares soaked with 0.5 μl 5-aza-cytidine (Sigma; 24 mM in H_2_O) or 2 μl Trichostatin A (Wako; 33 mM in DMSO) were placed in the center of the plate. After 5 hr at 32°C, 5 ml 0.7% top agar with 3 mg/ml Hygromycin B (Hyg; Hygrogold, InvivoGen) was overlaid for a final concentration of 0.5 mg/ml. For Basta selection, Vogel's salt solution with low nitrogen was used in all media [39]. For better imaging of smaller colonies on Basta selection plates we avoided top agar and instead 20 mg/ml Basta isolated from Rely (Bayer) [70] was poured in 5 ml of bottom agar, then 20 ml of non-selective media was overlaid for a final concentration of 4 mg/ml Basta. Plates were photographed after an additional 2 days of incubation at room temperature. To assay the effect of nicotinamide on expression of *hph *located at a telomere or at an interstitial location flanked by *am*^*RIP *^sequences, nicotinamide and Hyg were both added directly to agar medium and roughly 10^5 ^conidia were spread. Photographs were taken after 2 days of incubation at 32°C.

### Spot tests of mutants

To assay Hyg resistance, conidia were collected in water from flasks containing Vogel's minimal medium, 2% sucrose, 1.5% agar plus supplements following 1 week of growth at 32°C. Conidial suspensions were counted in a hemocytometer and serial dilutions were plated on Vogel's FGS agar medium with the same supplements, in the presence or absence of 1 mg/ml Hyg. Strains were allowed to grow for 2 days at 32°C. To test Basta resistance [39], conidia were prepared as above and plated on supplemented Vogel's FGS media prepared with low nitrogen and 1.5% agar in the presence or absence of 2 mg/ml Basta.

### Southern blots

Genomic DNA was isolated from 2-day cultures in Vogel's liquid medium as described [53]. Approximately 0.5 μg DNA was digested overnight with the designated restriction endonuclease and fractionated on 0.8% agarose gels. Transfer to nylon membranes and blotting were performed as described [53].

### Western blots

Nuclei were isolated as described [71] with the addition of HDAC inhibitors, TSA (1 μM, Wako) and sodium butyrate (50 mM, J.T. Baker). Nuclear proteins were fractionated by 10% SDS-PAGE. Following transfer to PVDF membrane (Millipore Immobilon-P) in 10 mM N-cyclohexyl-3-aminopropanesulfonic acid (CAPS), pH 11 with 20% methanol, blots were probed in phosphate-buffered saline (PBS) plus 3% non-fat dry milk with the following antibodies diluted as recommended by the manufacturer: α-H3 (Abcam ab1791), α-H3 acetyl K9 (Abcam ab4441), α-H3 acetyl K14 (Upstate 06–911), α-H3 acetyl K9/K14 (Upstate 06–599), α-H4 tetra-acetyl (Upstate 06–866) and α-H4 acetyl K16 (Upstate 07–329). Antibody detection was performed as described [25].

### Histone deacetylase assays

NST-1 was expressed as a GST fusion protein for *in vitro *activity assays. The *nst-1 *coding region was amplified with primers Nst1BamHI and Nst1NotI, digested with BamHI+NotI and cloned into BamHI+NotI-digested pGEX-5X-2 (GE Healthcare). The protein was expressed in *E. coli *strain BL21, induced with IPTG and purified on glutathione agarose (Sigma) in RIPA buffer (20 mM Tris-HCl (pH 7.5), 500 mM NaCl, 1% NP-40, 0.5% sodium deoxycholate, 1 mM DTT). Following elution with reduced glutathione, the GST fusion protein was dialyzed against RIPA buffer with 25% glycerol. *In vitro *activity assays were performed as described [15].

### Chromatin immunoprecipitation

ChIP experiments were performed as described [25] with the antibodies above and with α-H3 trimethyl K9 [72], α-H3 trimethyl K27 (Upstate 07–449), and α-H4 trimethyl K20 (Upstate 07–463). Primers used for the detection of precipitated DNA fragments are listed in Table 2. PCR reactions were repeated at least two times for each of two independent ChIP experiments.

## Competing interests

The authors declare that they have no competing interests.

## Authors' contributions

KMS helped design the study, generated strains, performed silencing assays, spot tests, Western blots, southern blots, northern blots, ChIP assays, sequence alignments and drafted and revised the manuscript. GOK helped design the study, created *nst *mutants, performed silencing assays, and revised the manuscript. CBM helped conceive of and design the study and generated *nst-1 *and telomere targeted marker strains. TKK generated strains and performed Southern blots. KKA generated the *nst-5 *mutant. MH helped generate *nst *mutants. MF helped conceive of and design the study and revised the manuscript. MRM performed the *in vitro *deacetylase assays. EUS conceived of and helped design the study, and revised the manuscript. All authors gave final approval of the manuscript.
